# Pretreatment Out-of-Pocket Expenses for Presumptive Multidrug-Resistant Tuberculosis Patients, India, 2016–2017

**DOI:** 10.3201/eid2605.181992

**Published:** 2020-05

**Authors:** Priya Rathi, Kalpita Shringarpure, Bhaskaran Unnikrishnan, Vineet Kumar Chadha, Vishak Acharya, Abirami Nair, Karuna D. Sagili, Suresh Shastri

**Affiliations:** Kasturba Medical College, Mangalore, Manipal Academy of Higher Education, Manipal, India (P. Rathi, B. Unnikrishnan, V. Acharya, A. Nair);; Medical College Baroda, Baroda, India (K. Shringarpure);; Central Leprosy Teaching and Research Institute, Tamil Nadu, India (V.K. Chadha);; National Tuberculosis Institute, Epidemiology and Research, Division Bengaluru, India (V.K. Chadha);; International Union against Tuberculosis and Lung Disease, South East Asia Office, New Delhi, India (K.D. Sagili);; Lady Willington State TB Centre, Bengaluru (S. Shastri)

**Keywords:** Multidrug-resistant tuberculosis, MDR TB, tuberculosis and other mycobacteria, presumptive MDR TB, direct cost, indirect cost, catastrophic cost, India, bacteria, respiratory infections, antimicrobial resistance, TB, tuberculosis

## Abstract

In India, under the National Tuberculosis Elimination Programme, the government provides free treatment for multidrug-resistant tuberculosis; however, many patients seek care elsewhere, which is costly. To determine those out-of-pocket expenses, we interviewed 40 presumptive patients and found that they spent more than their median annual income before registering for the government program.

In India, the annual economic loss resulting from tuberculosis (TB) is US $3 billion (*1*). Those in the economically productive age group (15–54 years) account for >70% of the total burden (*1*). Incidence of multidrug-resistant TB (MDR TB) is higher in India than anywhere else in the world; ≈99,000 new cases of MDR TB occur in India each year (*1*). Treatment of MDR TB is more complex, challenging, and costly to manage than that of drug-sensitive TB (*2*–*4*). In India, MDR TB is treated free of cost through programmatic management of drug-resistant TB (PMDT) under the National Tuberculosis Elimination Programme (*5*). However, most patients seek healthcare from the private sector and some resort to alternative forms of medicine, often preferring self-medication and consulting quacks over visiting the PMDT center (*6*,*7*). This behavior not only results in delayed diagnosis but also increases prediagnostic expenses (*7*). Increased expenses accompanied with loss of wages can compel patients and their families affected by TB to borrow money, take loans, or even sell their assets, thereby accentuating any existing financial crises in the family (*6*–*9*). Hence, we estimated the direct and indirect out-of-pocket expenses incurred for diagnosis and pretreatment evaluation by presumptive MDR TB patients in Mangalore, India.

## The Study

Mangalore is a coastal city in the state of Karnataka, India. The state has 6 PMDT centers. Presumptive MDR TB patients, when referred to PMDT centers, are subjected to drug sensitivity testing, preferably by use of a rapid molecular test (cartridge-based nucleic acid amplification assay), line probe assay, or culture, per PMDT guidelines (*10*). Those with an MDR TB diagnosis are admitted to the center for a week for pretreatment evaluation. All services provided under PMDT are free of cost to the patient (*10*).

**Figure 1:**
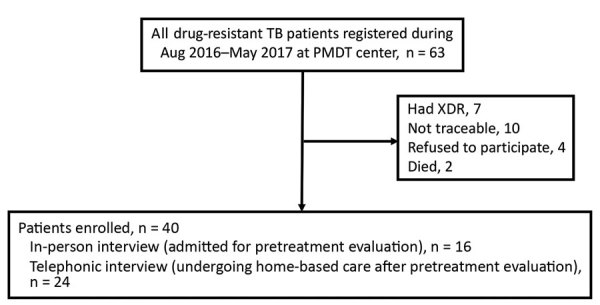
Flow chart showing patient enrollment in study of pretreatment out-of-pocket expenses for presumptive multidrug-resistant tuberculosis patients, India, 2016–2017.

We included in our study all adults (>15 years of age) with MDR TB who were registered under PMDT during August 2016–April 2017. By using a valid, pretested, semistructured tool, we interviewed patients about various costs incurred by themselves, their families, or both, from the time they became a presumptive MDR TB patient until they underwent pretreatment evaluation at PMDT. Information about various costs reported by patients was validated with bills, if available. We used the following cost categories: direct medical, direct nonmedical, indirect, and coping. Direct medical costs are expenses incurred during diagnosis and treatment of illness; direct nonmedical costs are costs of food, accommodations, and additional nutrition/supplements; indirect costs are the loss of wages because of illness; and coping costs are the costs of coping mechanisms (assets sold, school dropouts, loans, and money borrowed) (Appendix). Of the 40 MDR TB patients, the 16 who were admitted during the study period were interviewed in person and the 24 who continued home-based treatment were interviewed by telephone (Figure 1). Ethics approval was obtained from the Institutional Ethics Committee of Kasturba Medical College, Mangalore, and the Ethics Advisory Group of The International Union against Tuberculosis and Lung Disease, Paris, France.

Data were double entered in EpiData version 3.1 software (https://www.epidata.dk) and analyzed by using SPSS Statistics 25.0 (https://www.ibm.com) and EpiData analysis 2.2.2.183 software. Direct and indirect costs were summarized as median and interquartile ranges (IQRs). Categorical variables were expressed in proportions. Costs were collected by using Indian rupees (INR) converted to United States dollars (USD) based on the 2016 conversion rate (1 USD = 66.3731 INR). To compare the costs across different countries, we first converted the reported costs (USD) from other studies to local currency for the reported year, then adjusted them for inflation year by year until 2016 (*11*). Then we converted the costs back to USD by using the 2016 conversion rate (Appendix).

**Figure 2:**
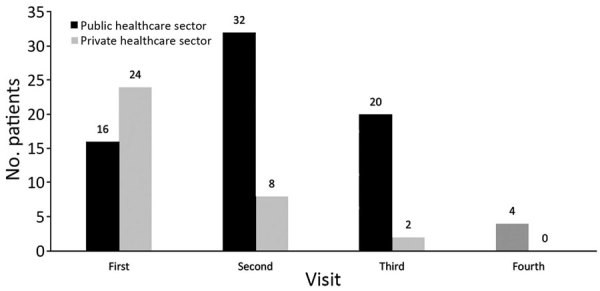
Distribution of visits to healthcare facilities in the public and private sectors by 40 presumptive multidrug-resistant tuberculosis patients before seeking care through programmatic management of drug-resistant tuberculosis, India, 2016–2017.

We included 40 of the 63 registered patients in the study. Median (IQR) age of participants was 39 (29–50) years. Most patients were male (28, 70%), and most lived in rural areas (28, 70%). Median (IQR) reported patient family income was $608 ($228–$912)/year. Of the 40 patients, 39 (97%) had pulmonary MDR TB and 24 (60%) had approached the private healthcare sector for their first clinical encounter (Table 1; Figure 2).

**Table 1:** Sociodemographic characteristics of 40 MDR TB patients treated at PMDT Centre, Mangalore, India, August 2016–April 2017*

Characteristic	No. (%)
Sex	
M	28 (70)
F	12 (30)
Education	
Illiterate	5 (12.5)
Primary school	15 (37.5)
Secondary school	14 (35.0)
Graduation/professional course	6 (15.0)
Type of occupation	
Salaried job	13 (32.5)
Daily wage	9 (22.5)
Business owner	5 (12.5)
Homemaker	8 (20.0)
Other†	5 (12.5)
Place of residence	
Urban	12 (30.0)
Rural	28 (70.0)
Socioeconomic status†	
Upper class	6 (15.0)
Upper-middle class	14 (35.0)
Middle class	8 (20.0)
Lower-middle class	7 (17.5)
Lower-class	5 (12.5)
Health facility sequence where MDR TB diagnosis made	
First	23 (57.5)
Second	14 (35.0)
Third	3 (7.5)
Fourth	0
Type of healthcare facility visited by patients before PMDT	
Private	24 (60)
Public	16 (40)

*MDR TB, multidrug-resistant TB; PMDT, programmatic management of drug-resistant TB; TB, tuberculosis. †Modified BG Prasad Classification (https://www.ijcmph.com/index.php/ijcmph/article/view/1242/1005).

The median (IQR) pretreatment out-of-pocket expenses incurred by patients were $171 ($72–$432) total, $105 ($49–$306) direct, and $51 ($2–$306) indirect. Within direct costs, direct nonmedical costs ($51) were more than direct medical costs ($37). Of the direct nonmedical costs, most was spent on food ($35). Most of the direct medical costs were for diagnostic investigation ($18) and treatment ($15) (Table 2).

**Table 2:** Median disaggregated costs incurred by 40 patients (households) from the stage of presumptive MDR TB to pre-MDR TB treatment evaluation, India, August 2016–April 2017*

Cost category	**Median (IQR), USD**
Total income	608.00 (228.00–912.00)
Total direct medical costs†	37.44 (7.10–198.24)
Total diagnosis, n = 38	01.58 (0.30–2.40)
Total investigation, n = 36	17.70 (3.19–60.27)
Total treatment, n = 26	15.07 (11.30–47.08)
Total admission, n = 15	45.20 (30.13–75.34)
Total direct nonmedical costs‡	51.20 (28.00–85.36)
Total food, n = 38	35.41 (18.08–64.97)
Total travel, n = 39	12.84 (5.73–12.84)
Total accommodations, n = 1	36.16 (36.16–36.16)
Additional nutrition, n = 38	01.51 (0.75–3.77)
Total direct costs§	105.12 (48.75–306)
Total indirect costs, n = 18¶	51.20 (1.60–306.00)
Total expenditures#	171.31 (72.00–432.00)
Total coping costs	640.00 (324.00–1,360)

*Because all patients did not incur all categories of costs, n differs for different categories. Median (IQR) is calculated only for those who incurred a given cost. IQR, interquartile range; MDR TB, multidrug-resistant TB; TB, tuberculosis: USD, US dollars. †Direct medical costs = sum of diagnosis investigation (general investigation and disease-specific investigations), complete blood count, erythrocyte sedimentation rate, liver function testing, renal function testing, spirometry, computed tomography, magnetic resonance imaging. Disease specific cost = sputum-smear microscopy, culture, drug-susceptibility testing, radiography, drugs, and hospitalization. ‡Direct nonmedical costs = sum of food, accommodation, travel by both patient and attendant. §Direct costs = sum of total direct medical costs and total direct nonmedical costs. ¶Indirect costs = loss of wages for patient and attendant during the visit.  #Total expenditure = sum of total direct and total indirect costs.

The median total pretreatment out-of-pocket expense incurred by patients in our study is similar to that found in a study in Peru ($210) after adjusting for inflation rate and cost conversion (*12*). The median direct out-of-pocket expenses are higher than the adjusted cost values found in previous comparable studies conducted in Ethiopia ($87), Indonesia ($47), and Peru ($67) and lower than that reported from Cambodia ($144) (*12*–*15*).

The median indirect out-of-pocket expense incurred by patients in India was $51 ($2–$306). This finding contrasts with those of studies in Ethiopia and Indonesia, where indirect pretreatment costs after adjustment for annual inflation were substantially lower (Ethiopia $9, Indonesia $8) (*15*).

In contrast, for patients in Ecuador, the adjusted direct out-of-pocket expenses were 5 times greater than those for patients in India ($105 vs. $549). The adjusted indirect expenses were 10 times greater ($51 vs. $578) (*12*) (Appendix Table).

In addition, 18 (45%) patients in the study lost their job because of the disease and had to borrow money for disease management and daily household needs before receiving accurate diagnosis and appropriate treatment. The percentages of persons with job losses were substantially lower than those reported for Peru (90%) and Ethiopia (72%) but similar to those for Indonesia (53%) (*12*,*15*).

Median coping cost incurred by patients in the study was $640 ($324–$1,360). Wingfield et al. reported a median debt of $435 and a loss of income of $2,450 before diagnosis for patients in Peru (*12*). In the study cohort, total median cost was $171 ($72–$432), which amounted to 28% of median total family income ($608). This expense, when combined with a coping cost of $640, resulted in a financial burden that was 1.25 times greater than the median total family income of the cohort ($608). Also, the cost of disease was $811 (sum of total median cost and median coping cost), and coping costs accounted for 79% of the total. Coping cost in a study conducted in Ecuador was as high as 7 times the average annual income (*14*). 

In our study, no patients reported school dropouts or separation in families. None of the patients reported selling assets such as property, gold, and other valuables. A total of 27 (67.5%) of the patients, approximately two thirds, had already incurred catastrophic expenses before they were registered for MDR TB treatment.

## Conclusions

Our study appraised the costs expended by MDR TB patients from a single PMDT center. Determination of a complete estimate of costs borne by all MDR TB patients in India would require a comprehensive study conducted at the community level and inclusion of patients receiving treatment from the public and private healthcare sectors.

New strategies that systematically engage private providers are needed to reduce the cost burden surrounding diagnosis for vulnerable patients. The government of India may consider widening the spectrum of free services before patient enrollment in a government-monitored treatment program channeled through the private sector.

Appendix Additional methods and results of study for pretreatment out-of-pocket expenses for presumptive multidrug-resistant tuberculosis patients, India, 2016–2017.
