# Supplementary material for: Pretreatment Out-of-Pocket Expenses for Presumptive Multidrug-Resistant Tuberculosis Patients, India, 2016–2017
Source: Emerg Infect Dis. 2020 May;26(5):989–92. doi: 10.3201/eid2605.181992 (PMC7181943; doi:10.3201/eid2605.181992)
Supplement: Appendix — Additional methods and results of study for pretreatment out-of-pocket expenses for presumptive multidrug-resistant tuberculosis patients, India, 2016–2017. [file 18-1992-Techapp-s1.pdf]

Article doi: https://doi.org/10.3201/eid2605.181992

Pretreatment Out-of-Pocket Expenses for Presumptive Multidrug-Resistant Tuberculosis Patients, India, 2016–2017

Appendix

Methods and Materials

Healthcare in India is majorly provided by government or public sector and private sector. Also there are alternative forms of medicine (i.e., Ayurveda, Yoga, Unani, Siddha, Homeopathy) and quacks, which people sort to for health related issues. The healthcare providers are majorly public health specialists who provides services under the national programs where they provide free of cost treatment and private practitioners who charge for their services. Health seeking behavior of the Indians are such that they first sort to self-medication, then alternative form of medicines and then to private practitioners (*1*,*2*). Those who can afford to pay continue the treatment in private sector. People in rural area and who cannot afford, avail services from public sector and healthcare programs, where treatment is provided free of cost or with negligible cost (*3*,*4*). RNTCP program is a stand-alone program where presently all forms of tuberculosis are managed by public sector itself. Multi-drug resistant TB is managed under PMDT (Programmatic Management of Drug-resistant Tuberculosis) (*5*,*6*).

Mangalore is a coastal city in the state of Karnataka. There are six PMDT centers in the state, one of them located in Mangalore. It caters to a population of around 100,000 including those from two neighboring districts and is responsible for pre-treatment evaluation and managing treatment for MDR-TB (*6*).

As per national PMDT guidelines, patients experiencing treatment failure or sputum smear positivity while on first-line anti-TB regimen and those previously treated with anti-TB drugs for ≥ 1 month are considered presumptive MDR TB patients (Glossary). They are subjected to drug sensitivity testing, preferably using a rapid molecular test (cartridge-based nucleic amplification assay test (CB-NAAT) or line probe assay (LPA) or culture. Presumptive

MDR TB cases are referred to PMDT centers. Persons with MDR TB are admitted to the PMDT center for a week for pretreatment evaluation at no cost to the patient (*5*,*6*).

This study was conducted as a part of output oriented operational research which was a time bound program. Therefore, we included all cases of adult MDR (≥ 15 years) registered under PMDT between August 2016 and April 2017 (9 months) in the study. The period of data collection was same as period of recruitment of patients in the study.

Assuming that XDR patient's pre-diagnostic and treatment trajectory is more complicated as compared to the MDR patient in India. Their number of visits to other Healthcare facilities prior to reaching PMDT center is more and hence the costs of Pre-diagnosis if included with MDRTB patients, may show an overestimation of expenditure. Hence, we decided to exclude them from study.

Information on various costs incurred was collected during an interview using a pre-tested, semi-structured tool. Costs incurred from the time they were presumptive MDR TB till the time they registered under PMDT and underwent pretreatment evaluation were collected with the help of study tool. The study tool was used to gather information on socio-demographic characteristics, reported income, various healthcare facilities (HCFs) visited, direct and indirect costs of diagnosis and pretreatment evaluation, and any potential coping mechanisms needed to maintain livelihood during this time (e.g., loans, selling off assets, and donations). Information of various costs reported by patients were validated with the bills, if available. Socio-economic status was measured using Modified BG Prasad classification (*7*). Those MDR-TB patients who were admitted during the study period were interviewed face-to-face (n = 16), while patients who continued home-based treatment were interviewed telephonically (n = 24). During face-to-face interview, if the patient was too ill to respond, one of the adult family members were interviewed as a proxy. For personal protection, the principal investigator (PI) wore an N-95 respirator and the patients wore surgical masks. Interviews occurred in an open space outside the PMDT Center with the PI sitting at an angle of 45 degrees from the patient.

Ethical approval was obtained from the Institutional Ethics Committee of Kasturba Medical College, Manipal University, Mangalore, and the Ethics Advisory Group of The International Union against Tuberculosis and Lung Disease, Paris, France.

Data analysis:

The data was double entered in EpiData version 3.1 software (EpiData Association, Odense, Denmark), matched for rectification if required and analyzed using SPSS (version 25.0) and EpiData analysis 2.2.2.183 software (*8*,*9*). The direct cost was further divided into direct medical and direct non-medical. The direct medical cost was calculated by simple addition of cost of consultancy, investigation, treatment. The direct non-medical was costs involved in transport, accommodation, food for both patient and attendant, if any. The indirect cost was summation of loss of wage or salary due to MDRTB. The direct and indirect costs were added to estimate total cost. Apart from these, the various coping mechanisms, adopted by the patients and their family to cover the medical and household expenses (for e.g., borrowing money, taking loan, selling assets, children's school dropouts) were included in calculating coping cost and were presented separately. Detailed definition and formula of calculation of different costs are mentioned in the Glossary. All forms of costs were summarized as median and Inter-Quartile Range (IQR). The socio-demographic characteristics and disease details were expressed in percentages. Costs were collected using Indian Rupees (INR) converted to United States Dollar (USD) amounts based on 2016 conversion rate (1 USD = 66.3731 INR) (*10*). We also compared our results with previously published studies that reported direct and indirect pretreatment expenditures. Because these studies occurred in different years and countries, for comparison, the reported costs were first converted to local currency, then were adjusted to inflation year by year till 2016 using following equation, $(E)i = (E)s * n=i-s-1(\pi)n=0 [\{1 + ((R)s+n/100) \}]$ (*11*). The inflated local currency was then converted back to USD using 2016 conversion rate for uniformity (*10*).

The median direct out-of-pocket expenditures (for examinations, laboratory testing, non-TB medications) for our patients was $105($49-306). This was substantially higher than the adjusted cost values of previous comparable studies conducted in Indonesia ($47), Peru ($67), and Ethiopia ($87), but lower than a report from Ecuador ($549) and Cambodia ($144). (*12*–*15*) The median total pretreatment out-of-pocket expenditure experienced by families in our study was $171($72-432). After adjustment, the total out-of-pocket expenditure costs in Cambodia ($394), and Peru ($210) were slightly lower, suggesting that indirect costs were an important component of overall pretreatment costs (*12*,*13*). Our results were in contrast to studies

conducted by van den Hof *et al.* in Ethiopia and Indonesia. They found substantially lower total median pretreatment costs after adjustment ($171 vs $95, and $55, respectively) (*14*). Variation in results could be due to methodological differences in data collection and analysis. van den Hof *et al.* reported low indirect cost in both country settings, whereas the results of Pichenda *et al.* in Ecuador found more than ten times adjusted indirect costs ($51 vs $578) and overall adjusted pretreatment costs ($171 vs $1126) as compared to our results (*13*,*15*). This could be attributed to steep rise in inflation rates in Ecuador (*16*,*17*).

Health seeking behavior among patients: We also found out the pattern of health seeking behavior of our patient. Majority (n=24, 60%) of the patients approached the private health care sector for the first clinical contact, while subsequently, proportion of patients visiting public health care sector rose.

Glossary of operational definitions used in the study

Presumptive MDR case (*6*): Any of the following: Presumptive DR-TB: It refers to the following patients in order of their risk: TB patients found positive on any follow-up sputum smear examination during treatment with first line drugs including treatment failures; TB patients who are contacts of DR-TB; previously treated TB patients; new TB patients with HIV co-infection; all notified new TB patients.

Quacks: Quacks are those practitioners who did not undergo a formal training in any form of medicine and do not hold any degree for the same.

Costs: All the costs defined below are borne by patients or/and their family members.

Direct Costs: Direct Cost = (direct medical cost + direct non-medical cost)

Direct Medical Cost: Sum of Diagnosis, investigation (general investigation and disease specific investigations), General Complete blood count, Erythrocyte Sedimentation Rate, Liver Function Test, Renal Function Test, spirometry Computer Tomography, Magnetic Resonance Imagine etc, Disease specific like sputum culture and microscopy, Drug Sensitivity Testing, X-ray etc., procedures, drugs and hospitalization prior to starting the treatment at DRTB center, if any).

Direct Medical Cost = Cost (diagnosis) + Cost (investigation) + Cost (treatment).

Direct Non-Medical Cost: Extra expenditure by the patient or his family for reasons other than diagnosis and treatment, like transportation, accommodation, purchase of food during visits to health facilities, extra nutrition.

Direct Non-Medical Cost = Cost (travel) + Cost (accommodation) + Cost (food)+ Cost (extra nutrition)

Indirect Cost: loss of wage / lost income /, loss of job for patient and the attendant if any from being labeled as presumptive MDR TB to pre-MDR TB treatment evaluation. This includes loss of wage /income of patient and attendant (if any) during the days where they could not go to work either due to hospital visit or days of incapacitation or for attendant while taking care of patient

Total Cost = Direct Cost + Indirect Cost

Coping Cost (*18*): Cost incurred if important household assets are sold off, children are taken out of school to contribute to household earnings, or loans with high interests are taken and donations

Coping Cost = Cost (assets sold) + Cost (school dropout) + Cost (loans with interest, if any) + Cost (money borrowed)

Disease Cost = Total Cost + Coping Cost

Reported Income: Sum total of household income as informed by the patient.

Catastrophic Cost (*19*): The WHO End TB Strategy suggests that any cost ≥20% of their total family income is considered as catastrophic and is associated with poor outcomes

Costs adjustment (*11*,*16*,*17*): Reported costs incurred by MDRTB patients from different countries were first adjusted to inflation within their current currency and then converted to USD for comparison.

Local currency inflation adjustment - Formula used to adjust for inflation in local currency-

(E)i = (E)start=s * n=i-s-1(π)n=0 [{1 + ((R)s+n/100) }] ,

where: S is the year of publication of study;(E)s is the reported cost by the study in the year of publication; i: year of current study (2016);(E)i is the cost of the study in current year (2016)

adjusted to inflation rate;(R)x is represents the inflation rate for year x. i.e (R)s+n is the inflation rate for year (s+n);(π): represents product of all values in the range of series.

The source of local inflation rates - World Bank. Official exchange rate. Inflation data . Available at: https://data.worldbank.org/indicator/PA.NUS.FCR.

The source of historical currency conversion - https://data.worldbank.org.

Formula used to adjust for inflation in local currency-

(E)i = (E)start=s * n=i-s-1(π)n=0 [{1 + ((R)s+n/100) }] , where: S is the year of publication of study;(E)s is the reported cost by the study in the year of publication; i: year of current study (2016);(E)i is the cost of the study in current year (2016) adjusted to inflation rate;(R)x is represents the inflation rate for year x. i.e (R)s+n is the inflation rate for year (s+n);(π): represents product of all values in the range of series.

Appendix Table. Adjusted out-of-pocket expenditures incurred by patients prior to anti-tuberculosis treatment in peer-reviewed publications in 2016.

Study	Country	Study Period	Cohort Size	Local currency conversion value for 1USD in the year of study*	Direct Out-of-pocket Pretreatment Expenditures Per Patient		Indirect Out-of-pocket Pretreatment Expenditures Per Patient		Total Out-of-pocket Pretreatment Expenditures per Patient	
					Reported value in USD	Comparable cost adjusted to 2016†	Reported value in USD	Comparable cost adjusted to 2016†	Reported value in USD	Comparable cost adjusted to 2016†
Wingfield T. et al, 2014	Peru	2002–2009	93	3.16	46	67	103	150	144	210
Pichenda K. et al, 2012	Cambodia	2008–2009	8	4000	95	144	144	218	260	394
Rouzier V. et al, 2010	Ecuador	2007	14	25000	374	549	394	578	768	1,126
van den Hof S. et al, 2016	Ethiopia	2013	169	18.28	68	87	7‡	9	75	95
van den Hof S. et al, 2016	Indonesia	2013	143	9636	39	47	7‡	8	46	55
Rathi P. et al, 2018 (Current Study)	India	2016–2017	40		105	105	51	51	171	171

*https://fxtop.com/en/historical-currency-converter.php,
†Adjusted for annual inflation rate of local currency for year 2016 and converted later to USD for comparison between countries. (Reference: †Inflation data from World Bank (http://api.worldbank.org/v2/en/indicator/FP.CPI.TOTL.ZG?downloadformat=excel).
‡Calculated as the median difference from the total out-of-pocket expenditures minus the direct out-of-pocket expenditures.

References

1. Samal J. Health seeking behaviour among tuberculosis patients in india: a systematic review. J Clin Diagn Res. 2016;10:LE01–06. PubMed https://doi.org/10.7860/JCDR/2016/19678.8598

2. Veesa KS, John KR, Moonan PK, Kaliappan SP, Manjunath K, Sagili KD, et al. Diagnostic pathways and direct medical costs incurred by new adult pulmonary tuberculosis patients prior to anti-tuberculosis treatment - Tamil Nadu, India. PLoS One. 2018;13:e0191591. PubMed https://doi.org/10.1371/journal.pone.0191591

3. Udwadia ZF, Moharil G. Multidrug-resistant-tuberculosis treatment in the Indian private sector: Results from a tertiary referral private hospital in Mumbai. Lung India. 2014;31:336–41. PubMed https://doi.org/10.4103/0970-2113.142101

4. Udwadia ZF, Pinto LM, Uplekar MW. Tuberculosis management by private practitioners in Mumbai, India: has anything changed in two decades? PLoS One. 2010;5:e12023. PubMed https://doi.org/10.1371/journal.pone.0012023

5. Ministry of Health and Family Welfare. Revised National Tuberculosis Control Program. National Guideline for Partnership, 2014. Central TB Division, Directorate General of Health Services, Nirman Bhawan, New Delhi: The Ministry. p. 99.

6. Ministry of Health and Family Welfare. RNTCP. Guidelines on Programmatic Management of Drug Resistant TB (PMDT) in India. New Delhi: The Ministry; 2016

7. Shaikh Z, Pathak R. Revised Kuppuswamy and B G Prasad socio-economic scales for 2016. Int J Community Med Public Health. 2017;4:997–9. https://doi.org/10.18203/2394-6040.ijcmph20171313

8. Lauritsen JM, Bruss M. Epidata Analysis. EpiData Command Processor tool. The EpiData Association, Odense Denmark, 2016. V2.2.2.183. [cited 2016 Dec 26]. https://tbrieder.org/epidata/course_abcde.pdf

9. Dean AG, Arner TG, Sunki GG, et al. Epi Info™, a database and statistics program for public health professionals [cited 2016 Dec 26]. https://www.cdc.gov/epiinfo/index.html

10. XE Corporation. Currency converter [cited 2018 Jun 26]. https://xe.com

11. Kumaranayake L. The real and the nominal? Making inflationary adjustments to cost and other economic data. Health Policy Plan. 2000;15:230–4. PubMed https://doi.org/10.1093/heapol/15.2.230

12. Wingfield T, Boccia D, Tovar M, Gavino A, Zevallos K, Montoya R, et al. Defining catastrophic costs and comparing their importance for adverse tuberculosis outcome with multi-drug resistance: a prospective cohort study, Peru. PLoS Med. 2014;11:e1001675. PubMed https://doi.org/10.1371/journal.pmed.1001675

13. Pichenda K, Nakamura K, Morita A, Kizuki M, Seino K, Takano T. Non-hospital DOT and early diagnosis of tuberculosis reduce costs while achieving treatment success. Int J Tuberc Lung Dis. 2012;16:828–34. PubMed https://doi.org/10.5588/ijtld.11.0688

14. Rouzier VA, Oxlade O, Verduga R, Gresely L, Menzies D. Patient and family costs associated with tuberculosis, including multidrug-resistant tuberculosis, in Ecuador. Int J Tuberc Lung Dis. 2010;14:1316–22. PubMed

15. van den Hof S, Collins D, Hafidz F, Beyene D, Tursynbayeva A, Tiemersma E. The socioeconomic impact of multidrug resistant tuberculosis on patients: results from Ethiopia, Indonesia and Kazakhstan. BMC Infect Dis. 2016;16:470–7. PubMed https://doi.org/10.1186/s12879-016-1802-x

16. Leive A, Xu K. Coping with out-of-pocket health payments: empirical evidence from 15 African countries. Bull World Health Organ. 2008;86:849–56. PubMed https://doi.org/10.2471/BLT.07.049403

17. World Health Organization. The end TB strategy [cited 2017 Jul]. http://www.who.int/tb/End_TB_brochure.pdf

18. The World Bank. Official exchange rate. Inflation data [cited 2019 Oct 9]. https://data.worldbank.org/indicator/PA.NUS.FCR

19. The World Bank. Official exchange rate [cited 2020 Jan 1]. https://data.worldbank.org/indicator/PA.NUS.FCRF
